# Antimicrobial resistance patterns and clinical outcomes in ventilator-associated pneumonia

**DOI:** 10.6026/973206300212410

**Published:** 2025-08-31

**Authors:** Urvashi Krunalkumar Sharma, Atit Dineshchandra Shah

**Affiliations:** 1 Department of Microbiology, Dr. N D Desai Faculty of Medical Science and Research, Dharmsinh Desai University, Nadiad, Gujarat, India; 2 Department of Microbiology, Smt. N.H.L Municipal Medical College, Ahmedabad, Gujarat, India

**Keywords:** Bacterial pathogens in VAP, antimicrobial drug susceptibility, multidrug resistance, prognosis

## Abstract

Ventilator-associated pneumonia (VAP) poses a major challenge in ICUs due to its high morbidity, mortality, and rising antimicrobial resistance. This observational study assessed microbial isolates, resistance patterns, and outcomes in 321 VAP cases out of 472 ventilated patients in a tertiary care hospital. *Acinetobacter baumannii* complex (33.33%) was the most common pathogen, with 97.01% showing multidrug resistance. The overall mortality rate among VAP patients was 45.79%. These findings highlight the urgent need for effective antibiotic stewardship and infection control strategies in ICU settings.

## Background:

Healthcare-associated pneumonia (HCAP) is the second most frequent hospital-acquired infection and there are reported 15-20 % of hospital-acquired infections and it creates a big impact on patient outcomes, patient stays, and the health care system [1]. The most dangerous of them is ventilator-associated pneumonia (VAP), a life-threatening complication that is triggered by the condition of the patients who have to be put on the ventilation machine which is usually caused by critical illness (respiratory failure). Mechanical ventilation is a necessary and life-saving procedure which, however, leaves its recipients with a significantly heightened risk of lower respiratory tract infection with the mortality rates varying between 20-40% on the basis of underlying conditions and the time of diagnosis and treatment [2]. VAP is characterized as pneumonia that takes place within 48 to 72 hours of endotracheal intubation, and it shows clinical, radiologic, and microbiologic signs of infection [3]. Besides being subjected to the complications of high morbidity and associated mortality, it also is one of the causes of long stays in hospitals, use of more antimicrobials, and high costs of healthcare. Geographically and temporal microbiological landscape of VAP is not uniform, but in many cases dominated by multidrug resistant (MDR) gram negative bacilli, *i.e.* *Acinetobacter baumannii*, *Klebsiella pneumoniae*, and *Pseudomonas aeruginosa*, further complicating the control of these infections [4]. Local epidemiology and trends in resistance of VAP pathogens are crucial to developing an effective formulation and personalization of antimicrobial therapy and effective empirical treatment protocols. Earlier reports have emphasized that regular monitoring of the trends of antimicrobial susceptibility should be carried out to counter the emergence of the MDR organisms in the ICUs [5]. Therefore, it is of interest to comprehensively characterize the microbial profile and antimicrobial susceptibility patterns of causative pathogens related to ventilator associated pneumonia (VAP) cases in our intensive care unit (ICU) environment.

## Materials and Methods:

This is an observational study conducted at the Department of Microbiology, Smt. N.H.L. Municipal Medical College and Ahmedabad from March'2019 to February' 2020. 321 patients developed VAP (pneumonia was developed more than 48 h after intubation) out of 472 mechanically ventilated patients, were included in the study. The patients satisfied all required criteria were diagnosed with VAP.

## Inclusion criteria:

[1] Patients admitted in Intensive Care Unit or transferred to the unit from other medical or surgical wards

[2] Patients kept on mechanical ventilation for >48 hours

## Exclusion criteria:

[1] Patients already have pneumonia at the time of ICU admission

[2] Patients who develop pneumonia in the first 48 hours of mechanical ventilation

Under strict aseptic precautions, samples (endotracheal aspirates) were collected from the patients and transported immediately to the laboratory in appropriate settings; samples were inoculated on Nutrient agar, MacConkey agar and Blood agar plates and incubated aerobically for 24 hours at 37°c or for 48 hours if needed. Growth and cultural characteristics were observed the next day. Identification and AST were performed by automation. Furthermore, Identification of isolates was confirmed by conventional method also.

## Identification of bacterial pathogens and their antimicrobial susceptibility testing:

AST cards (N280 and N281) and identification cards were inoculated with suspensions of microorganisms. After being put into the loading chamber, the cards were sealed and placed in a revolving carousel set at 37° degrees Celsius. After that, they were taken out of the carousel and brought to the optical system for data gathering and reaction reading.

## Quality control:

The Vitek 2 compact machine is validated using the standard strain of *Escherichia coli*, Klebsiella pneumonia, and Staphylococcus aureus as per the manufacturer's instructions. During the study period, the control strain was checked every 15 days.

## Results:

A total 472 patient admitted in our hospital ICUs during study period from March 2019 to February 2020. Out of these, 321 patients fit in definitions of VAP (68%), while 151 considered as non-VAP (32%) pneumonia (Table 1 - see PDF). Total of 487 tracheal aspirates were collected from these 321 patients. From 487 samples, 30 samples were negative in culture while 457 were positive in culture signifying very high rate of isolations of organism from tracheal specimen from ICU patients. 603 different microorganisms were isolated from 457 tracheal samples (Table 2 - see PDF). Various organisms isolated from VAP (total 603) were Gram negative bacilli (n=595, 98.67%) (NFGNB isolated 335/595, 56.30%), Gram positive cocci (n=6, 0.99%) and Candida spp. (n=2, 0.33%). The highest number of bacterial isolates were found *Acinetobacter baumannii* complex (n=201, 33.33%), followed by *Klebsiella pneumoniae* (n=170, 28.19%), *Pseudomonas aeruginosa* (n=117, 19.40%), *Escherichia coli* (n=38, 6.30%) and others (Table 3 - see PDF). *Acinetobacter baumannii* complex showed highest sensitive to colistin (96.46%) followed by tigecycline (78.79%), minocycline (47.49%), amikacin (21.01%), trimethoprim-sulfamethoxazole (11.11%), cefoperazone-sulbactam (60.6%), gentamicin (5.05%), cefepime (3.53%), levofloxacin (2.79%), imipenem (2.02%), meropenem (2.02%), piperacillin-tazobactam (2.02%), ciprofloxacin (2.02%), doripenem (1.68%), ticarcillin-clavulanic acid (1.68%). Highest sensitivity was observed in *K. Pneumonia* to colistin (88.80%) followed by amikacin (32.54%), gentamicin (29.59%), tigecycline (26.63%), imipenem (26.04%), trimethoprim-sulfamethoxazole (24.85%), ertapenem (23.75%), meropenem (22.49%), minocycline (22.22%), cefoperazone-sulbactam (20.71%), piperacillin-tazobactam (14.20%), cefepime (12.42%), amoxicillin-clavulanic acid (11.88%), levofloxacin (11.89%), ciprofloxacin (10.1%). Pseudomonas showed the highest sensitivity to colistin (90.43%) followed by gentamicin (52.14%), cefepime (48.72%), amikacin (48.72%), ceftazidime (44.25%), ciprofloxacin (43.97%), cefoperazone-sulbactam (42.02%), doripenem (34.51%), piperacillin-tazobactam (33.62%), imipenem (31.90%), levofloxacin (31.30%), meropenem (30.17%), ticarcillin-clavulanic acid (10.71%). *E. coli* showed highest sensitive to colistin (100%), minocycline (100%) followed by tigecycline (92.10%), amikacin (76.32%), doripenem (66.67%), gentamicin (63.16%), imipenem (60.53%), ertapenem (60%), meropenem (57.90%), trimethoprim-sulfamethoxazole (42.10%), cefoperazone-sulbactam (36.84%), piperacillin-tazobactam (21.05%), amoxicillin-clavulanic acid (17.14%), cefepime (15.79%), levofloxacin (2.94%), cefuroxime (2.86%).

All 6 gram-positive organisms (Staphylococcus species) were 100% sensitive to daptomycin, linezolid, teicoplanin and vancomycin. A higher rate of sensitive pattern was noted to clindamycin (83.33%), rifampicin (83.33%) and tetracycline (83.33%). 4 out of total 6 staphylococcus spp. were observed as MRS (methicillin resistant staphylococcus) and 2 were methicillin sensitive (Table 4 - see PDF). A total number of patients developed VAP (321) showed Death (n=147, 45.79%), Discharge (n=118, 36.76%) and DAMA (n=56, 17.45%). Out of 147 total deaths, 67 (n=143, 46.85%) death were seen in early onset VAP and 80 (n=178, 44.94%) were seen in late onset VAP. It was observed that a greater number of deaths seen in early onset VAP (Table 5 - see PDF). Out of 513 MDR microorganisms, 195 *Acinetobacter baumannii* complex (n=201, 97.01%),20 Proteeae family (n=21, 95.24%),159 *K. Pneumonia*e (n=170, 93.53%), 35 *E. coli* (n=38, 92.1%), 22 *S. marcescens* (n=25, 88%), 4 Enterobacter group (n=6, 66.67%), 71 *P. aeruginosa* (n=117, 60.68%) and 7 other non-fermenters (n=17, 41.17%) were isolated. Most common multidrug resistant organism was *Acinetobacter baumannii* complex followed by *K. Pneumonia*e and *P. aeruginosa* (Table 6 - see PDF). Multidrug resistant microorganisms were resistant to a majority of antibiotics in all groups of antibiotics showed 100 % resistance to aztreonam, ceftazidime, cefuroxime, doripenem, levofloxacin, ticarcillin/Clavulanic acid in majority microorganisms.

## Discussion:

Endotracheal secretions were sent for sensitivity testing, bacteriological culture and identification in order to help prevent VAP by starting and adjusting antibiotic therapy appropriately, which would result in a positive outcome. The majority of our tertiary care institute's critical patients is terminally ill and come from other hospitals; they may need artificial ventilation, which could cause difficulties or lengthen their hospital stay. Thus, a crucial step for clinicians is choosing the right antibiotic therapy for treatment and an increased chance of MDR organism formation may result from inappropriate therapy. VAP rate in present study was compared with studies of Ranjan *et al.* [4] (57.14%) and Dey *et al.* [5] (45.4%) (Table 7 - see PDF). *Acinetobacter baumannii* complex (33.33%) was the predominant isolate in early and late onset of VAP and chief causative agent for VAP followed by *Klebsiella pneumoniae* (28.19%), *Pseudomonas aeruginosa* (19.40%) and others (Table 3 - see PDF). Similar distribution of microorganisms was seen in study of Sopia *et al.* [6] and isolated different microorganisms were compared with various studies of Tripathi *et al.* [7] and Petdachai *et al.* [8] in (Table 8 - see PDF). Because of the warm, humid environment that encourages infection, these organisms are especially prevalent in hospital settings. They can therefore colonise patient mucosa and different device surfaces. These organisms also have a survival advantage because they produce biofilm, which shields them from hospital drugs. In a study by Dey *et al.* [5] from Manipal, the commonest microorganism causing both early and late onset VAP was Acinetobacter spp. (48.94%) followed by *Pseudomonas aeruginosa* (25.53%). Sensitivity of *Acinetobacter baumannii* complex and *K. Pneumonia*e in present study was compared with the study of Sopia, *et al.* [6] in which sensitivity seen in Acinetobacter species were tigecycline (100%), colistin (100%), piperacillin/tazobactam (66.66%), ceftazidime (5.55%) and *K. Pneumonia*e were sensitive to colistin (100%), tigecycline (100%), piperacillin/tazobactam (70.58%), imipenem (64.7%), gentamicin (11.11%), amikacin (5.55%). In the study of Goel *et al.* [9], *Acinetobacter baumannii* showed 100% resistance to ceftazidime, 87% resistance to Amikacin, 89% resistance to Ciprofloxacin and in *K. Pneumonia*e, 95.5% resistance in ciprofloxacin, 63.6% resistance is Amikacin.

AST pattern of *P. aeruginosa* was compared with the study of Goel *et al.* [9] showed sensitivity to meropenem (77.2%), amikacin (39.6%), ceftazidime (31.6%). AST of *E. coli* was compared to the study of Pradhan *et al.* [10] showed *E. coli* sensitive to amikacin (100% in micu and 85.7% in sicu), imipenem (66.7% in micu and 92.9% in sicu), meropenem (66.7% in micu and 85.7% in sicu), piperacillin/tazobactam (100%) and ciprofloxacin (100%). In present study, drugs like colistin (100%), tigecycline (92.10%), imipenem (60.53%), ertapenem (60%), amikacin (76.32%) showed more susceptible For *Escherichia coli* than Klebsiella pneumonia [colistin (88.80%), tigecycline (26.63%), imipenem (26.04%), ertapenem (23.75%) and amikacin (32.54%)]. Overall sensitivity of colistin observed in *Acinetobacter baumannii* complex (96.41%) and in *E. coli* (100%) and tigecycline sensitivity (78.46% in *Acinetobacter baumannii* complex and 92.10% in *E. coli*) except klebsiella pneumonia, which showed 88.68% sensitivity to colistin and 22.01% to tigecycline. Ventilator-associated pneumonia (VAP) is an important cause of healthcare-associated infections, resulting in prolonged hospitalization with increased morbidity and mortality [11]. The incidence of MDR A. baumannii and *P. aeruginosa* had been found to be 37.5% and 40% respectively in VAP patients by Golia *et al.* [12]. MDR A. baumannii showed high level of resistant to carbapenems. Carbapenem resistance Acinetobacter was reported to be about 75% among VAP isolates in study of Gurjar *et al.* [13]. MDR pattern of A. baumannii and *P. aeruginosa* was compared to the study of Goel *et al.* [14] in which 100% A. baumannii isolates was multidrug resistant, showed resistant to ciprofloxacin (92.59%), amikacin (92.89%), imipenem (88.89%), ceftazidime (85.18%), piperacillin/tazobactam (37.04%) and *P. aeruginosa* showed resistant to gentamicin (100%), aztreonam (88.23%), ciprofloxacin (82.35%), amikacin (82.35%), imipenem (47.06%), ceftazidime (35.29%), piperacillin/tazobactam (23.53%). Overall carbapenem resistance in MDR isolates in the present study found to be 80%-100%. It was compared to the study of Tran *et al.* [15] in which Acinetobacter was resistant to all antibiotics including imipenem (93%), meropenem (90%), ertapenem (100%), ceftriaxone (95%), ciprofloxacin (90%), cefepime (94%), ceftazidime (93%) and piperacillin (95%). In our study, non-fermenters were the most common etiological agents isolated from VAP from ICU patients. This observation in AST pattern of non-fermenters (*Acinetobacter baumannii* complex and *Pseudomonas aeruginosa*) was compared in the study of Goel *et al.* [9] reported that 40% *P. aeruginosa* was resistant to all the antibiotics used against it and meropenem sensitive 77.2% followed by piperacillin-tazobactum (50.5%). Other investigators had reported a lower rate of resistance in various drug resistances [6].

Multidrug resistant microorganisms were common in intensive care settings. The antimicrobial susceptibility pattern of isolates obtained in the present study showed that most gram-negative bacilli were multidrug resistant including *Acinetobacter baumannii* complex, Klebsiella pneumonia, *Pseudomonas aeruginosa* and *E. coli*. such a high level of drug resistance had also been documented in studies conducted by Azzab *et al.* [16] and Dey *et al.* [5]. The antibiotic susceptibility profile of MDR microorganism is alarming in the present study. The incidence of MDR isolates was found to be high (82.21%) in the present study, which indicated the need for appropriate empirical antibiotic treatment effective against MDR organisms. All gram-positive isolates were 100% sensitive to vancomycin, teicoplanin and linezolid but out of them 50% were resistant to methicillin. It was also compared to the study Azzab *et al.* [16]. In the present study, it was found that the mortality rate among these patients was (45.79%). It had been seen that the mortality was high in early onset VAP (46.85%). In studies undertaken by Panwar *et al.* [17] and Mukhopadhyay *et al.* [18], mortality rates were found to be 37% and 61.9% respectively. Other studies had shown that VAP associated with mortality was 13% [19]. Ventilator-associated pneumonia (VAP) is frequently caused by gram-negative bacteria, with *Acinetobacter baumannii*, *Klebsiella pneumoniae*, and *Pseudomonas aeruginosa* being the most prevalent pathogens across multiple hospital settings. A notable concern is the high rate of multidrug resistance among these organisms, which poses significant challenges to treatment and necessitates reliance on last-resort agents like colistin. These findings emphasize the critical importance of local antimicrobial surveillance and the formulation of empirical antibiotic protocols tailored to prevailing resistance trends [11, 20. These variations in mortality rates could be explained by differences in patient characteristics, inadequate and improper antimicrobial treatment and increase length of mechanical ventilation and duration of hospital stay, antimicrobial resistance of the organism responsible, severity of illness, co-morbid factors and host response factors. The study population and number of isolates in present study may not represent the present scenario, further studies would be needed to strengthen the outcome of the present study and help clinicians to initiate antibiotics.

## Conclusion:

*Acinetobacter baumannii*, *Klebsiella pneumoniae*, and *Pseudomonas aeruginosa* were the predominant pathogens in VAP, with a high prevalence of multidrug resistance. The associated mortality rate was notably high, especially in early-onset cases. Strengthening antibiotic stewardship and infection control is essential to reduce VAP burden in ICUs.
